# The IFN-stimulated gene IFI27 counteracts innate immune responses after viral infections by interfering with RIG-I signaling

**DOI:** 10.3389/fmicb.2023.1176177

**Published:** 2023-04-28

**Authors:** Laura Villamayor, Darío López-García, Vanessa Rivero, Luis Martínez-Sobrido, Aitor Nogales, Marta L. DeDiego

**Affiliations:** ^1^Department of Molecular and Cell Biology, Centro Nacional de Biotecnología (CNB-CSIC), Madrid, Spain; ^2^Texas Biomedical Research Institute, San Antonio, TX, United States; ^3^Center for Animal Health Research, CISA-INIA-CSIC, Madrid, Spain

**Keywords:** IFI27, innate immune responses, interferon, inflammation, influenza, SARS-CoV-2, virus-host interactions

## Abstract

The recognition of viral nucleic acids by host pattern recognition receptors (PRRs) is critical for initiating innate immune responses against viral infections. These innate immune responses are mediated by the induction of interferons (IFNs), IFN-stimulated genes (ISGs) and pro-inflammatory cytokines. However, regulatory mechanisms are critical to avoid excessive or long-lasting innate immune responses that may cause detrimental hyperinflammation. Here, we identified a novel regulatory function of the ISG, IFN alpha inducible protein 27 (IFI27) in counteracting the innate immune responses triggered by cytoplasmic RNA recognition and binding. Our model systems included three unrelated viral infections caused by Influenza A virus (IAV), Severe Acute Respiratory Syndrome coronavirus 2 (SARS-CoV-2), and Sendai virus (SeV), and transfection with an analog of double-stranded (ds) RNA. Furthermore, we found that IFI27 has a positive effect on IAV and SARS-CoV-2 replication, most likely due to its ability to counteract host-induced antiviral responses, including *in vivo*. We also show that IFI27 interacts with nucleic acids and PRR retinoic acid-inducible gene I (RIG-I), being the interaction of IFI27 with RIG-I most likely mediated through RNA binding. Interestingly, our results indicate that interaction of IFI27 with RIG-I impairs RIG-I activation, providing a molecular mechanism for the effect of IFI27 on modulating innate immune responses. Our study identifies a molecular mechanism that may explain the effect of IFI27 in counterbalancing innate immune responses to RNA viral infections and preventing excessive innate immune responses. Therefore, this study will have important implications in drug design to control viral infections and viral-induced pathology.

## Introduction

Some pathogen molecules, such as lipopolysaccharides, glycoproteins, proteoglycans and nuclear acid motifs, such as double-stranded RNA (ds) RNA and 5′-phosphate single stranded RNA (ssRNA) motifs, are known as pathogen-associated molecular patterns (PAMPs), and are essential components to initiate an innate immune response upon recognition by pattern recognition receptors (PRRs) (Wilkins and Gale, 2010; Jensen and Thomsen, 2012). There are different types of PRRs, including Toll-like receptors (TLRs), and retinoic acid-inducible gene I (RIG-I)-like receptors (RLRs). RLRs are a family of RNA sensors localized in the cytosol, and comprise three members: RIG-I, melanoma differentiation-associated protein 5 (MDA-5) and laboratory of genetics and physiology 2 (LGP2) (Wilkins and Gale, 2010; Jensen and Thomsen, 2012; Rehwinkel and Gack, 2020). TLRs, such as TLR-3, as well as MDA-5 and RIG-I, are important for the recognition of viral infections. All RLRs encode a central helicase domain and a carboxy-terminal domain (CTD). These two domains work together to detect immunostimulatory RNAs, such as viral RNAs. RIG-I and MDA5 additionally comprise two amino-terminal caspase activation and recruitment domains (CARDs), which mediate downstream signal transduction. LGP2 lacks the CARDs and is widely believed to regulate RIG-I and MDA5 (Wilkins and Gale, 2010; Jensen and Thomsen, 2012; Rehwinkel and Gack, 2020). Signal transduction converges in the activation of several transcription factors like interferon-regulatory factors 3 and 7 (IRF-3 and IRF-7, respectively), and nuclear factor κB (NF-κB) (Jensen and Thomsen, 2012; Rehwinkel and Gack, 2020). These transcription factors regulate the expression of type I and III interferons (IFNs), and inflammatory cytokines, which are essential in innate immune responses against different pathogens (Jensen and Thomsen, 2012; Rehwinkel and Gack, 2020).

IFN molecules bind their receptors in neighbour cells activating a signaling cascade through the Janus kinase transducer and activator of transcription (JAK–STAT) pathway (Schneider et al., 2014). Thus, IFNs are able to activate a second round of autocrine and paracrine signals, that allows infected and surrounding cells to activate anti-viral programs mediated by Interferon Stimulated Genes (ISGs) (Kikkert, 2020). Hundreds of ISGs have already been described, and they have an enormous variety of functions (Schneider et al., 2014). Many ISGs have a clear antiviral function, but there are also ISGs that are able to prevent an excessive immune response, which can be deleterious to the host, playing a regulatory role (Komuro et al., 2008; Richards and Macdonald, 2011; Chathuranga et al., 2021). As an example, we previously described the molecular mechanisms underlying negative modulation of IFN responses mediated by the ISGs IFI6, IFI44 and IFI44L that facilitate virus replication (DeDiego et al., 2019a,b; Villamayor et al., 2023).

Although ISGs have been extensively studied, there are still many genes whose function(s) are not clearly defined, for instance, IFI27 (also known as ISG12a), which was first described in 1993 (Rasmussen et al., 1993). This gene encodes a putative 122 amino acid hydrophobic protein of 12 kDa, that has an N-terminal mitochondrial targeting sequence (Cheriyath et al., 2011). It belongs to the FAM14 family, comprising four genes in humans (IFI6 or G1P3, IFI27 or ISG12a, IFI27L2 or ISG12b and IFI27L1 or ISG12c) (Cheriyath et al., 2011). Both IFI6 and IFI27 are small hydrophobic proteins sharing 36% overall amino acid sequence homology (Sajid et al., 2021). Several lines of evidence support that IFI27 acts as a pro-apoptotic factor since it is associated or present at the mitochondrial membrane, and it contributes to IFN-dependent perturbation of the mitochondrial membrane permeability (Rosebeck and Leaman, 2008), it sensitizes cells for TNF*α* and the BH3 mimetic gossypol induced apoptosis (Gytz et al., 2017), and it augmented TNF-related apoptosis-inducing ligand (TRAIL)-induced apoptosis through intrinsic apoptotic pathway (Liu et al., 2019). Furthermore, there are several studies that demonstrate that IFI27 plays a role in viral infections, with either negative or positive effects on viral disease progression depending on the study and type of infection. High IFI27 levels have been detected in blood of infants hospitalised with Respiratory Syncytial Virus (RSV) (Fjaerli et al., 2006). Furthermore, higher levels of IFI27 have been associated with more severe cases, more requirements of mechanical ventilation, more frequent hospitalization, and longer hospital stays in preterm infants infected with RSV, proposing the use of IFI27 as a biomarker of RSV disease severity and outcome (Gao et al., 2021). IFI27 expression is also upregulated in the blood of patients suffering influenza infection (Tang et al., 2017; Ravi et al., 2022), and IFI27 upregulation has been shown to be able to discriminate between influenza and bacterial respiratory infections (Tang et al., 2017). Furthermore, IFI27 expression is upregulated in the blood of SARS-CoV-2 positive patients (Gupta et al., 2021; Huang et al., 2021) and allows to distinguish non-infected from SARS-CoV-2-infected subjects (Gupta et al., 2021). IFI27 has been proposed as a possible therapeutic target in HIV infections, since it has been found that expression of IFI27 was upregulated in HIV-positive patients and downregulated in patients receiving antiretroviral therapy (Huang et al., 2022).

On the other hand, IFI27 has shown a non-apoptotic antiviral effect against Hepatitis C Virus (HCV) (Xue et al., 2016). IFI27 mediates the ubiquitin-dependent degradation of HCV NS5A protein, as it is able to promote the association of the S-phase kinase-associated protein 2 (SKP2), which is an ubiquitin E3 ligase, to HCV NS5A. Consequently, IFI27 acts as a crucial adaptor that promotes SKP2 to interact with HCV NS5A and mediates its degradation via the proteasome (Xue et al., 2016).

These findings suggest that IFI27 plays several roles that may depend on the types of viral infections and diseases. However, it has not been clearly defined its biological importance as well as the mechanisms by which IFI27 participates in the innate immune response as an ISG during viral infection. In this line, we show, for the first time, that IFI27 expression facilitates IAV and SARS-CoV-2 viral replication. Furthermore, we report a completely novel function for IFI27 in negatively modulating innate immune responses in cell cultures and mice. The molecular mechanism on IFI27’s downregulation of innate immune responses involves the interaction of IFI27 to RNA, enabling interaction with RIG-I and impairing RIG-I activation and downstream innate immune responses. Therefore, IFI27 provides a negative feed-back mechanism that counteracts excessive inflammatory responses to RNA viral infection.

## Materials and methods

### Cells

Human embryonic kidney 293T (ATCC CRL-11268), human lung epithelial carcinoma A549 (ATCC CCL-185), and African green monkey kidney epithelial Vero E6 (ATCCCRL-1586) cells, were kindly provided by Prof. Luis Enjuanes (Centro Nacional de Biotecnología, CNB-CSIC, Spain). Madin-Darby Canine Kidney (MDCK) epithelial cells (ATCC CCL-34). All cells were grown at 37°C in air enriched with 5% CO_2_ using Dulbecco’s modified Eagle’s medium (DMEM, Gibco) supplemented with 10% fetal bovine serum (Gibco), and 50 μg/mL gentamicin (Gibco). A549 cells overexpressing human ACE-2 (hACE-2, A549-ACE-2) were grown in the same media containing 2.5 μg/mL of blasticidin (ThermoFisher Scientific).

### Viruses and virus titrations

Virus stocks of IAV A/Puerto Rico/8/1934 H1N1 (PR8) (wt and recombinant viruses) were grown in MDCK cells (51). All IAV infections were performed in the presence of 1 μg/mL of tosylsulfonyl phenylalanyl chloromethyl ketone (TPCK)-treated trypsin (Sigma). IAV was titrated by immunofocus assay (fluorescent focus units, FFU/mL), in confluent MDCK cells seeded in 96-well plates, as previously described (Nogales et al., 2014). SeV, Cantell strain (Kochs et al., 2007), was grown in embryonated chicken eggs. The recombinant Vesicular Stomatitis Virus, Indiana strain, encoding the green fluorescent protein, GFP (rVSV-GFP) (Stojdl et al., 2003), and the SARS-CoV-2 (kindly provided by prof. Luis Enjuanes, at Centro Nacional de Biotecnología, CNB-CSIC, Spain) were grown in Vero E6 cells. rVSV-GFP and SARS-CoV-2 were titrated by plaque assay (plaque forming units, PFU/mL) in confluent monolayers of Vero E6 cells seeded in 24-well plates, as previously described (DeDiego et al., 2016; Saiz et al., 2021).

### Plasmids

Polymerase II expression pCAGGS plasmids encoding IFI27 (GenBank accession number NM_001130080.3) C-terminally fused to an HA epitope tag (pCAGGS-IFI27-HA), and PRKRA (Protein Activator of Interferon Induced Protein Kinase EIF2AK2) fused to a FLAG epitope tag (pCAGGS-PRKRA-FLAG, GenBank accession number NM_003690.5) were generated by RT-PCR using total RNA isolated from human epithelial A549 cells and cloned using standard techniques (primers available upon request). pCAGGS plasmids expressing RIG-I protein (GenBank accession number AF038963.1) fused to a FLAG epitope tag (pCAGGS-RIG-I-FLAG) and a pCAGGS plasmid expressing GFP, (pCAGGS-GFP) were previously described (Mibayashi et al., 2007). pMP31 plasmid encoding mitochondrial antiviral signaling protein (MAVS) fused to a FLAG epitope tag (pMP31-MAVS-FLAG) was obtained from Addgene, and previously described (Patel et al., 2012).

To generate IFI27 KO cells, RNA guides (gRNA) were selected using the webpages.^1^ The short guide RNA (sgRNA) sequence selected was: 5′- GTGCCATGGGCTTCACTGCGG-3′. The cDNAs complementary to the two different sgRNAs were cloned in the pX330 plasmid (kindly provided by Dr. Pedro A. Mateos, Universidad de Alcalá de Henares, Spain), expressing the RNA guides under the U6 promoter and encoding the CAS9 gene and a gene encoding for resistance to puromycin. To this end, a pair of forward and reverse oligonucleotides for the generation of each sgRNA (IDT) were annealed and phosphorylated by incubating the forward and reverse primers with T4 polynucleotide kinase (New England Biolabs), during 30 min at 37°C, followed by 95°C during 5 min and then ramp down to 25°C, at 5°C/min. The phosphorylated and annealed primers were inserted into plasmid vector pX330 between BbsI restriction sites.

To generate a plasmid containing two different 2A autoproteolytic cleavage sites and the NS1 and NEP genes, the previously described pDZ-NS-2xBsmBI plasmid (Nogales et al., 2015), which contains the NS1 ORF, without the stop codon or splice acceptor site, and two BsmBI sites followed by the porcine teschovirus-1 (PTV-1) 2A autoproteolytic cleavage site (ATNFSLLKQAGDVEENPGP) and NEP (Nogales et al., 2015). An inverse PCR using primers 5´-AATTACGCGTGGAGAGGGCAGAGGAAGTCTGCTAACATGCGGTGACGTCGAGGAGAATCCTGGACCTGGGTCCGGCTGAGACGAGATCTC-3′ and 5´-AATTACGCGTTCCAACTTCGCTTCTAATTGTTCCCGCCATTTCTCG −3′ was used to introduce the thosea asigna virus (TAV) 2A autoproteolytic cleavage site (EGRGSLLTCGDVEENPGP). The final plasmid, named pDZ-NSsplit2xBsmBI-2A, contains the following elements: 5′-non-coding region (NCR)/NS1/link (GTRG)/TAV-2A/GSG-BsmBI/BglII/BsmBI-GSG/PTV-1 2A/NEP/3′-NCR. For generating recombinant IAV-IFI27 and IAV-mCherry, plasmids pDZ-NSsplit-2xBsmBI-2A-IFI27 and pDZ-NSsplit-2xBsmBI-2A-mCherry were generated. Briefly, IFI27 and mCherry were amplified by PCR using specific primers flanked by BsmBI restriction sites and the amplified PCR products were digested with BsmBI and used to clone in the pDZ-NSsplit2xBsmBI-2A plasmid.

### Generation of A549 IFI27 KO cells

A549 cells (12-well plate format) were transfected with the pX330 plasmid expressing the sgRNA, using lipofectamine 3000 (ThermoFisher Scientific, 1250 ng/well). At 24 h post-transfection (hpt), cells were treated with 1 μg/mL of puromycin (InVivoGen), for selecting the cells transfected with the plasmids. At 48 h after the puromycin treatment, media was exchanged with fresh media without puromycin. Surviving cells were detached with trypsin and the cells were cloned three times by limiting dilution. Different clones were genotyped by sequencing (Macrogen). To generate A549 cells susceptible to SARS-CoV-2 infection, parental and IFI27 KO A549 cells were transduced with a retrovirus expressing hACE-2 and a gene conferring resistance to blasticidin, kindly provided by Dr. Pablo Gastaminza (CNB-CSIC).

### Knock-down of IFI27 using siRNAs

Human A549 or 293T cells (24 or 96-well plate format) were transfected independently with two different “silencer select” small interfering RNAs (siRNAs) specific for human IFI27 (ThermoFisher Scientific, s7139 and s194542), or with the non-targeting (NT) negative control (ThermoFisher Scientific, AM4635), twice, 24 h apart. All siRNAs were transfected at a final concentration of 20 nM, using lipofectamine RNAiMax (ThermoFisher Scientific), according to the manufacturer’s instructions.

### IFN response assays

Human A549 and 293T cells were transfected with siRNAs specific for IFI27, or the NT control siRNA for 24 h. Alternatively, parental A549 cells and cells specifically knocked-out for IFI27 were seeded. Then, A549 cells were infected with IAV (multiplicity of infection, MOI 1), A549-hACE-2 cells were infected with SARS-CoV-2 (MOI 1), or 293T cells were infected with SeV (MOI 3) for 24, and/or 48 h. Alternatively, A549 cells were transfected with 60 ng/ml of polyinosinic-polycytidylic acid (poly(I:C), Sigma) using polyethylenimine (PEI, Polysciences) during 24 h, or the cells were treated with human IFN-alpha hybrid protein (universal type I IFN, PBL assay Science) (Horisberger and de Staritzky, 1987; Felgenhauer et al., 2020). IAV and SARS-CoV-2 titers were determined as described above. Alternatively, total RNA was extracted, and RT-qPCRs were performed, as described below. In addition, A549 IFI27 KO cells were seeded and transfected with 60 ng/mL of poly(I:C) using PEI. At 16 h after treatment, cells were infected with rVSV-GFP for 24 h and viral titers in cell culture supernatants were determined in Vero cells as previously described (DeDiego et al., 2016).

### RT-qPCR

mRNA levels of IFI27, IFN*λ*1, and IFN-induced protein with tetratricopeptide repeats 2 (IFIT2) in human A549, and 293T cells, were analyzed. To this end, total RNAs were extracted using the total RNA extraction kit (Omega Biotek). Retrotranscriptase (RT) reactions were performed using the High Capacity cDNA transcription kit (ThermoFisher Scientific) at 37°C for 2 h, using random primers, and total RNA as template. qPCRs were performed using TaqMan gene expression assays (Applied Biosystems) specific for human IFI27 (Hs01086373_g1), human IFIT2 (Hs00533665_m1), human IFNλ1 (Hs00601677_g1), human CXCL10 (Hs00171042_m1), and human GAPDH (Hs02786624_g1) genes. Quantification was achieved using the threshold cycle (2^−ΔΔ*CT*^) method (Livak and Schmittgen, 2001) and normalized with GAPDH expression levels.

### Western blots

Cells were lysed in Co-IP buffer (NaCl 250 mM; EDTA 1 mM; 50 mM TrisHCl, pH 7.5; NP-40 0.5%) containing protease (ThermoFisher Scientific) and phosphatase inhibitors (Merck) inhibitors. Cell lysates were mixed with Laemmli sample buffer containing 2.5% *β*-mercaptoethanol, and heated at 95°C for 5 min, before SDS-PAGE electrophoresis. Proteins were transferred to nitrocellulose membranes (Biorad), and detected using primary rabbit polyclonal antibodies (pAbs) specific for HA tag (Sigma Aldrich H6908), and IFI27 (St John’s laboratory STJ190336), and mouse monoclonal antibodies (mAbs) against the FLAG tag (Sigma-Aldrich F3165), and GFP (Merck 11814460001); following by incubation with a 1:4,000 dilution of goat anti-rabbit (pAb) or anti-mouse (mAb) IgG antibodies conjugated to horseradish peroxidase (Sigma-Aldrich). Membranes were revealed by chemiluminescence, according to the manufacturer’s recommendations, using the SuperSignal west femto maximum sensitivity substrate (ThermoFisher Scientific).

### Virus rescue

Virus rescue was performed as previously described (Clark et al., 2017; Nogales et al., 2018, 2021). Briefly, co-cultures of 293T and MDCK cells (1:1 proportion) in 6-well plates were co-transfected with 1 μg of the ambisense WT pHW-PB2, -PB1, -PA, -HA, -NP, -NA and-M of IAV-PR8 plasmids together with the NS split plasmids encoding non-overlapping NS1 and NEP genes plus IFI27 (IAV-IFI27), or mCherry (IAV-mCherry), using lipofectamine 3000 (ThermoFisher Scientific). At 16 hpt, medium was replaced with DMEM containing 0.3% BSA, antibiotics and 1 μg/mL of TPCK-treated trypsin (Sigma). At 48 h, cell culture supernatants were collected and used to infect fresh MDCK cells. At 72 hpi, recombinant viruses were plaque purified and a stock was generated by infecting (MOI of 0.001) MDCK cells. Stocks were titrated by immunofocus assay as previously described (Nogales et al., 2014). The identity of the recombinant viruses was confirmed by sequencing (Macrogen).

### *In vivo* experiments

C57BL/6 6-week-old female mice were purchased from Envigo and maintained at the vivarium from the National Center for Biotechnology in a pathogen-free environment. Procedures involving animals were approved by the CSIC ethics committee for animal experimentation and by the Division of Animal Protection of the regional government of Madrid in compliance with national and European Union legislation (PROEX89.5/20). Mice were slightly anesthetized with isoflurane and then, intranasally inoculated with 2,000 FFU of the recombinant viruses per mice. Viral titers in the lungs at 24 and 48 hpi (*n* = 4 per group) were determined. To this end, mice were sacrificed and the right lung lobules were extracted and homogenized. Virus titers were determined by immunofocus assay on MDCK cells as indicated above. In addition, levels of IFIT2, IFNL3, TNF, and CCL2 mRNAs were analyzed in the lungs at 24 and 48 hpi. For this, the left lung lobules were extracted and incubated in RNAlater (Ambion) at 4°C during 24 h prior to adding the lungs to RNA lysis buffer, and homogenizing the lungs manually using a dounce homogenizer. Total RNA was extracted from homogenized lungs using the total RNA kit (Omega Biotech). RT reactions were performed at 37°C, during 2 h using the high capacity cDNA transcription kit and random hexamers (ThermoFisher Scientific) to generate the cDNAs. qPCRs were performed using Taqman gene expression assays (Applied Biosystems) specific for the murine CCL2 (Mm00441242_m1), IFIT2 (Mm00492606_m1), TNF (Mm00443258_m1), IFNL3 (Mm00663660_g1), and GAPDH (Mm99999915_g1) genes. Data from qPCR was assessed following threshold cycle (2^-ΔΔ*CT*^) methodology (Livak and Schmittgen, 2001) and normalized with GAPDH expression levels.

### Binding of IFI27 to poly(I:C)

Human 293T cells (6-well plate format) were transiently transfected with plasmids expressing IFI27, GFP, and PRKRA (pCAGGS-IFI27-HA, pCAGGS-GFP, and pCAGGS-FLAG-PRKRA, respectively), using lipofectamine 3000 (ThermoFisher Scientific). At 24 h, cells were lysed in Co-IP buffer and cellular extracts were bound to poly(I:C)-conjugated agarose beads. To prepare poly(I:C)-conjugated agarose beads, 6 mg of poly(C)-conjugated agarose beads (Sigma) per sample were washed five times with Tris-Buffered Saline (TBS) buffer (25 mM Tris, 150 mM NaCl). Beads were then resuspended in buffer containing 50 mM Tris and 50 mM NaCl and incubated overnight with 120 μg of inosinic acid (Sigma). Beads were washed twice with TBS, resuspended in TBS buffer containing 1 mM EDTA and 0.5% Triton X-100, and incubated at 4°C for 3 h with the cellular extracts expressing IFI27, GFP, or PRKRA. The mixture was washed 4 times with TBS buffer containing 1 mM EDTA and 0.1% Tween 20, and the bound proteins were eluted in loading buffer at 95°C during 5 min. The eluted proteins were analyzed by Western blotting using antibodies, as described above.

Alternatively, human 293T cells were transiently transfected with the pCAGGS plasmid expressing IFI27, using lipofectamine 3000 (ThermoFisher Scientific) for 24 h. Then, cells were transfected with biotinylated poly(I:C) (Invivogen) or with poly(I:C) without biotin, as control, at 200 ng/mL. At 24 h after treatment, cells were lysed in Co-IP buffer containing protease and phosphatase inhibitors, and the clarified cellular extracts were incubated with agarose beads bound to streptavidin (ThermoFisher Scientific), during 4 h, at 4°C. The samples were washed 4 times with TBS buffer containing 1 mM EDTA and 0.1% Tween 20, and the bound proteins were eluted in loading buffer at 95°C during 5 min. The eluted proteins were analyzed by Western blotting using antibodies, as described above.

### Immunoprecipitation assays

Human 293T cells (100 mm-plate format) were transiently co-transfected with the plasmids pCAGGS-IFI27-HA alone or together with pCAGGS-RIG-I-FLAG, using lipofectamine 3000 (ThermoFisher Scientific), for 24 h. The total amount of transfected DNA plasmid was maintained constant by co-transfecting the empty pCAGGS plasmid when needed. Later, cells were transfected with poly(I:C) (1,000 ng/ml) using PEI for an additional 24 h or were infected with SeV (MOI 3) during 24 h, and cells were lysed in the Co-IP buffer and cleared by centrifugation. Where indicated, cellular lysates were treated with RNaseA (10 U/ml), RNase T1 (400 U/ml) and RNAse III (10 U/ml), during 30 min at 37°C, as previously reported (Laraki et al., 2008). Cleared cell lysates were incubated overnight at 4°C with the anti-FLAG affinity resin (Sigma-Aldrich, A2220). Then, the mixtures were washed three times in TBS buffer containing 0.1% SDS, and precipitated proteins were dissociated using 0.1 M glycine buffer at pH 2.4, denatured in loading buffer and incubated at 95°C, during 5 min. Then, samples were analyzed by electrophoresis and Western blot as described above using anti-HA (IFI27), and anti-FLAG (RIG-I) specific Abs.

### Immunofluorescence and confocal microscopy

Confluent monolayers of human 293T cells were grown on sterile glass coverslips (24-well format) and were transiently transfected, using lipofectamine 3000 (ThermoFisher Scientific), with the pCAGGS plasmids expressing RIG-I-FLAG and IFI27-HA. At 24 hpt, cells were transfected with poly(I:C). Alternatively, confluent monolayers of MDCK cells were infected (MOI 0.1) with IAV-mCherry or IAV-IFI27. At 24 h after poly(I:C) transfection or at 24 hpi, cells were fixed and permeabilized with 10% formaldehyde and 0.1% Triton-X100 during 20 min at RT. Then, cells were blocked with 2.5% BSA in PBS and RIG-I-FLAG, IFI27-HA and viral NP were detected with mouse anti-FLAG, and rabbit anti-HA polyclonal antibodies, and with a mouse antibody for viral NP (mAb HB-65), respectively. Coverslips were washed 4 times with PBS and stained with secondary anti-mouse and anti-rabbit Abs conjugated to Alexa Fluor 488 and 546 (Invitrogen), and nuclei were stained using DAPI (ThermoFisher Scientific), during 45 min at RT. Coverslips were mounted in ProLong Gold antifade reagent (Invitrogen) and analyzed on a Leica STELLARIS 5 confocal microscope. Images were acquired with the same instrument settings and analyzed using the Fiji software.

### RIG-I and MAVS overexpression assays

To analyze the induction of innate immune responses mediated by RIG-I, human 293T cells (24-well plate format) were transfected with the pCAGGS plasmids expressing the IFI27-HA, and RIG-I fused to a FLAG tag during 24 h. As control, 293T cells were transfected with the pCAGGS plasmids expressing the IFI27-WT, and MAVS fused to a FLAG tag during 24 h. Alternatively, the 293T cells were transfected with two siRNAs specific for human IFI27 (ThermoFisher Scientific, s7139 and s194542) and with the plasmid expressing RIG-I-FLAG. Where indicated, 24 h after transfections, the cells were infected with SeV (MOI 3) for an additional 24 h. Total RNAs were extracted using the total RNA extraction kit (Omega Biotek), and the expression of IFNL1 was analyzed by RT-qPCR, as specified above.

## Results

### Induction of IFI27 expression after poly(I:C) transfection and IAV and SARS-CoV-2 infections

**Figure 1:**
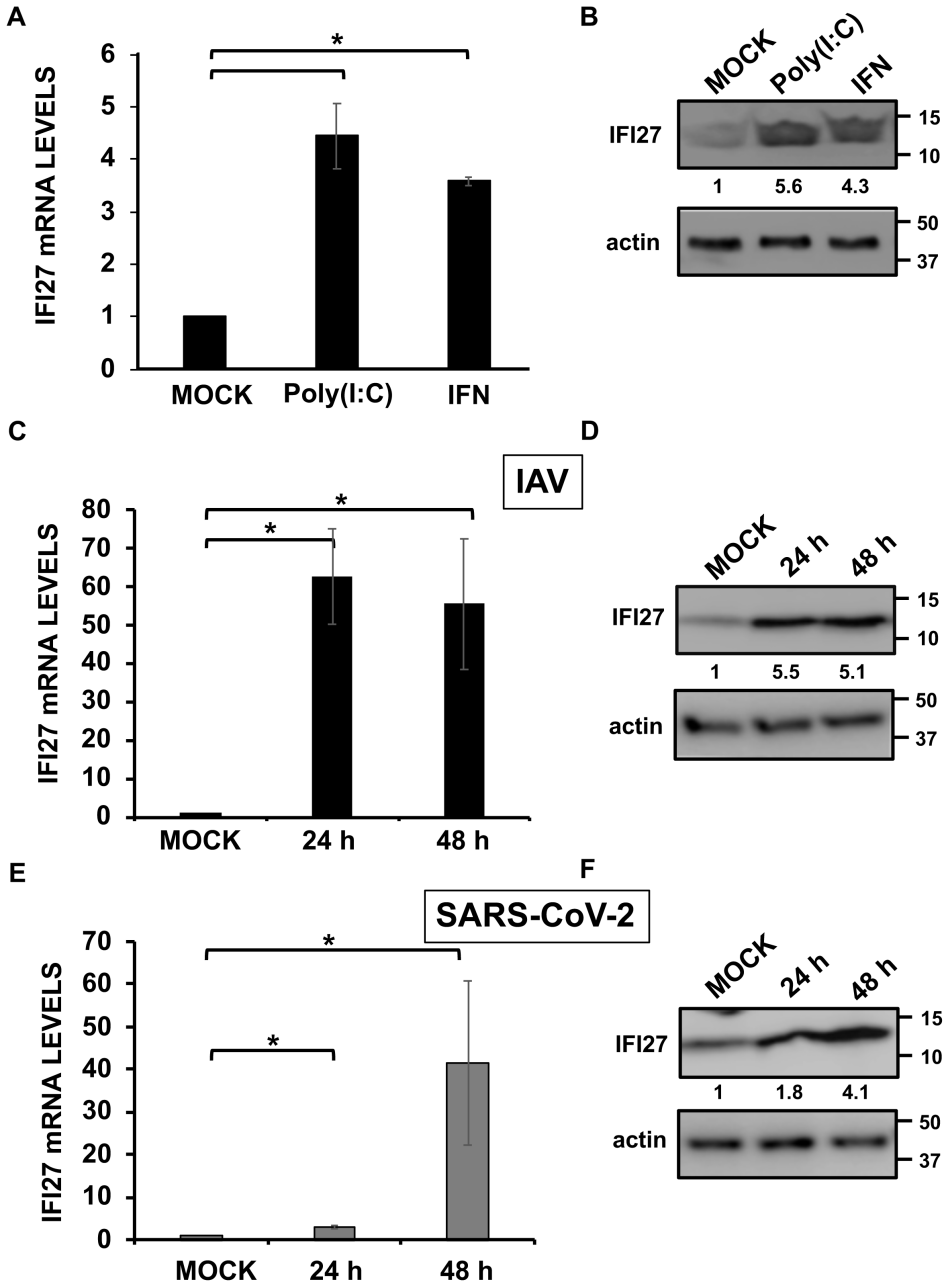
IFI27 expression is upregulated by poly(I:C) transfection, IFN treatment, and IAV and SARS-CoV-2 infection. **(A,B)** A549 cells were transfected with poly(I:C) (60 ng/mL) or treated with recombinant universal type I IFN (600 units/mL) during 24 h. **(C,D)** A549 cells were infected with IAV (MOI 1) during 24 and 48 hpi (black columns in **C**). **(E,F)** A549-hACE-2 cells overexpressing hACE-2 were infected with SARS-CoV-2 (MOI 1) during 24 and 48 hpi (gray columns in **E**). **(A,C,E)** IFI27 gene expression was evaluated by RT-qPCR and compared to the levels in non-treated or non-infected cells. Error bars represent standard deviations (SD) of results of measurements performed in triplicate wells. *p < 0.05 using a Student’s *t* test. **(B,D,F)** Cellular extracts were subjected to Western blotting using antibodies specific for IFI27 and actin, as loading control. Western blots were quantified by densitometry using ImageJ software, and the amounts of IFI27 were normalized to the amounts of actin (numbers between the IFI27 and actin blots).

IFI27 expression is induced by type I IFNs in most, if not all, IFN-responsive cells, as shown by previous studies (Kelly et al., 1986; Porter et al., 1988; Rasmussen et al., 1993; Parker and Porter, 2004; Liu et al., 2007; Cheriyath et al., 2011). In addition, and supporting these results, it has been shown that type I IFN-mediated induction of IFI27 expression is highly impaired in cells deficient for IFNAR1, encoding a subunit of the type I IFN receptor (Crowl and Stetson, 2018). To confirm these findings, human A549 cells were transfected with poly(I:C), as an analog of dsRNA that is produced during the replication of many RNA viruses, or treated with recombinant IFN-α (Horisberger and de Staritzky, 1987; Felgenhauer et al., 2020), and the levels of IFI27 mRNA and protein expression were measured by RT-qPCR and Western blot, respectively. In both cases, levels of IFI27 mRNA were induced by 4.5 and 3.7-fold (Figure 1A), while levels of endogenous IFI27 protein were induced by 5.6 and 4.3-fold, after poly(I:C) transfection and recombinant IFN-α treatment, respectively (Figure 1B), indicating that IFI27 behaves as an ISG in human A549 cells.

IFI27 expression is upregulated after IAV and SARS-CoV-2 infections in patient’s blood cells (Zhai et al., 2015; Tang et al., 2017; Gupta et al., 2021; Huang et al., 2021; Ravi et al., 2022), SARS-CoV-2-infected, patient’s respiratory swabs (Mick et al., 2020), and IAV-infected cell cultures (Ioannidis et al., 2012). To confirm that IFI27 expression is also induced after IAV and SARS-CoV-2 infections, same human A549 cells were infected with influenza A virus (IAV, MOI 1). In addition, we infected with SARS-CoV-2 (MOI 1) A549 cells overexpressing human ACE-2 protein (A549-hACE-2), so that the cells become susceptible to SARS-CoV-2, as previously reported (Cagno, 2020). IFI27 expression was upregulated by 60-and 3-fold at 24 h post-infection (hpi), and by 55 and 40-fold at 48 hpi, after IAV and SARS-CoV-2 infections, respectively (Figures 1C,E), indicating that IFI27 expression is also induced during IAV and SARS-CoV-2 infections. Accordingly, as determined by Western blot, endogenous IFI27 protein levels were also upregulated during IAV and SARS-CoV-2 infections by 5.1 and 4.1-fold, at 48 hpi, respectively (Figures 1D,F), indicating that IFI27 behaves as an ISG in human A549 cells.

### Effect of IFI27 on viral replication

**Figure 2:**
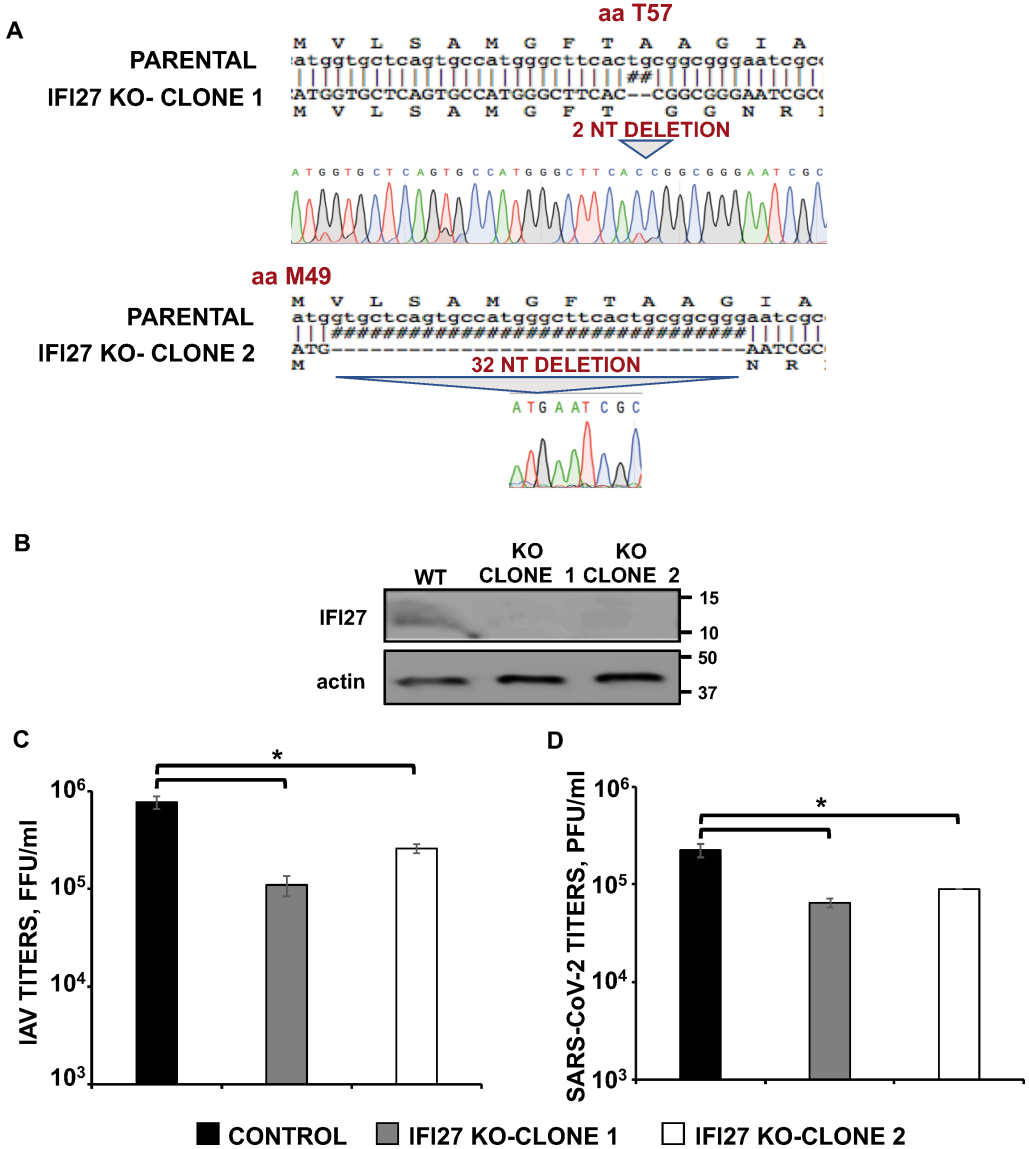
IFI27 expression affects IAV and SARS-CoV-2 replication. **(A)** Sequencing of A549 parental cells and IFI27 A549 KO cells, showing the deletion of 2 and 32 nucleotides within the open reading frame of IFI27 (bottom), in comparison to parental cells (top). The amino acid positions at which the deletions occurred in the two clones, respectively (T9 and M1) are indicated. **(B)** The expression of IFI27 and actin (as control) was evaluated in parental and IFI27 KO cells treated with poly(I:C), by Western blot using antibodies specific for these proteins. **(C)** The two different clones of A549 cells specifically KO for the IFI27 gene were infected with IAV. Viral titers in cell culture supernatants were measured at 24 h post-infection (hpi) by immunofocus assay in canine MDCK cells (fluorescence forming units per ml, FFU/mL) and compared to the titers in the parental, control cells. **(D)** A549 clones specifically KO for IFI27 and overexpressing hACE-2, were infected with SARS-CoV-2. Viral titers in cell culture supernatants were measured at 48 hpi, by a plaque assay in Vero E6 cells (plaque forming units per ml, PFU/mL), and compared to the titers in the parental, control cells. Three different experiments were performed, with similar results. Data are represented as the mean and standard deviations of triplicate measures. *p < 0.05 (for comparisons between parental IFI27 KO cells and control cells using Student’s *t* test).

Next, human A549 knock-out (KO) cells for IFI27 were generated using CRISPR/Cas9 technologies to analyze the cellular roles of IFI27 and whether IFI27 modulates viral replication. To this end, we used a plasmid encoding an sgRNA, the gene for CAS9, and a gene encoding resistance to puromycin to select the transfected cells in the presence of this antibiotic. After obtaining different clones by cell limiting dilution, two clones were selected for further studies. The two clones encoded 2 and 32-nucleotide deletions, respectively, leading to a frameshift starting from amino acid 9 and 2 of the protein, respectively (Figure 2A). Importantly, we confirmed by Western blot, that endogenous IFI27 protein was not expressed in the two IFI27 KO clones treated with poly(I:C), whereas the protein was expressed in the parental A549 cells treated with poly(I:C) (Figure 2B).

To study the effect of IFI27 on IAV and SARS-CoV-2 infections, parental and IFI27 KO A549 cells were infected with IAV, PR8 strain (MOI 1), and the production of extracellular infectious viruses was evaluated at 24 hpi. IAV titers decreased between 3 and 8-fold in IFI27 KO A549 cells, compared to parental A549 cells (Figure 2C). Furthermore, parental and IFI27 KO A549-hACE2 cells were infected with SARS-CoV-2 (MOI 1), and viral titers were analyzed. SARS-CoV-2 titers decreased approximately 3 times in the IFI27 KO A549 cells, in comparison to parental A549 cells (Figure 2D). These data showed that IFI27 it should read positively correlates, instead of negatively, as follows: These data showed that IFI27 expression positively correlates with viral production negatively correlates with viral production and that this effect is not specific for a given virus.

**Figure 3:**
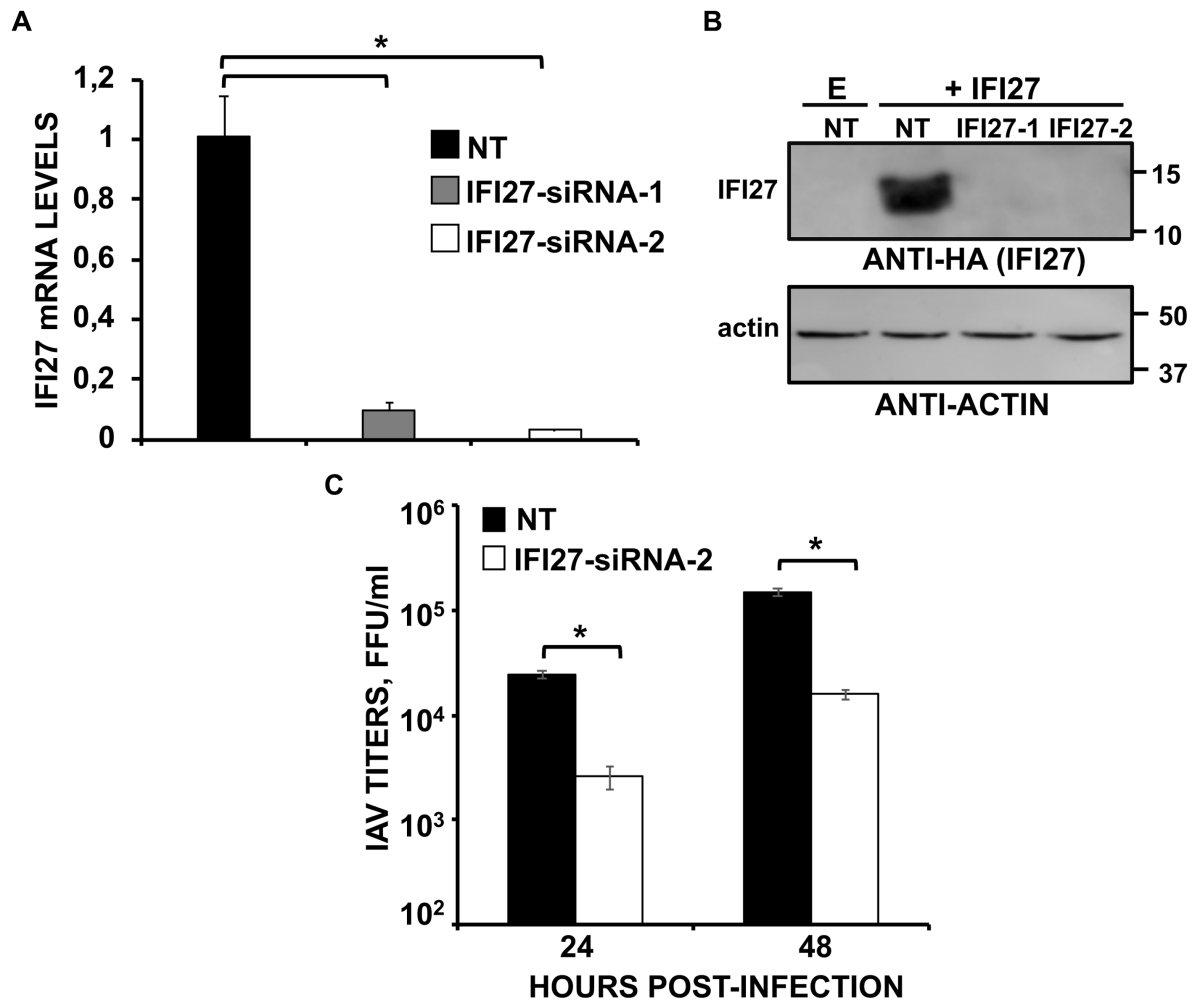
Knocking-down IFI27 expression negatively affects IAV infection. **(A–C)** Human A549 cells were transfected with non-targeted (NT) or IFI27 siRNAs during 24 h. **(A)** Later on, total RNAs were purified and mRNA levels for IFI27 were assessed by RT-qPCR. **(B)** Cells were transfected for 24 h with the plasmid expressing IFI27 fused to an HA tag or with an empty plasmid, as control. IFI27 expression and actin expression (as control) was analyzed by Western blot using anti-HA antibodies (to detect IFI27; top) and anti-actin antibodies (bottom). Molecular weight markers are indicated (in kDa) on the right. **(C)** At 24 hpt, cells were infected with IAV. Cell culture supernatants were collected at 24 and 48 hpi and titrated by immunofocus assay. Three different experiments were performed, with similar results. *p < 0.05 (for comparisons between control and IFI27 knocked-down cells at 24 and 48 hpi using Student’s *t* test).

To confirm these results using another experimental approach, human A549 cells were knocked-down for IFI27 expression using two different siRNAs specific for IFI27. A non-targeted (NT) siRNA was used as control. At the mRNA level, the expression of IFI27 was silenced by more than 90% in the cells transfected with the two siRNAs specific for IFI27, as compared to the control cells (Figure 3A). To ascertain that IFI27 expression was also knocked-down at the protein level, cells were transfected with the IFI27 siRNAs, or the NT siRNA control, and after 24 h the cells were transfected with the pCAGGS plasmid encoding IFI27 fused to an HA tag, or empty pCAGGS. At 24 h post-transfection, Western blot analysis demonstrates IFI27 expression in A549 cells transfected with the NT siRNA, but not in cells transfected with the two IFI27 siRNAs (Figure 3B). Therefore, the IFI27 siRNAs downregulated IFI27 expression at both, the mRNA and protein levels. After IFI27 gene silencing, the cells were infected with IAV to analyze whether IFI27 expression positively affects viral production. Viral titers at 24, and 48 hpi decreased by 8-fold in the cells silenced for IFI27, compared to the control cells (Figure 3C). These results confirm that IFI27 silencing negatively affects IAV infection, even in a context in which we use a WT virus encoding the viral non-structural protein 1 (NS1), which encodes a RIG-I antagonistic activity (Guo et al., 2007; Mibayashi et al., 2007; Opitz et al., 2007).

### Effect of IFI27 on antiviral responses

**Figure 4:**
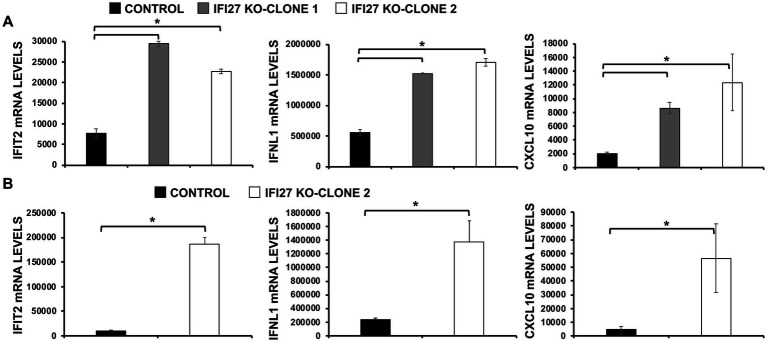
IFI27 negatively modulates innate immune responses induced by viral infections. **(A)** Two clones of IFI27 KO A549 cells were infected with IAV during 24 h. **(B)** One clone of A549 cells specifically KO for the gene IFI27 was infected with SARS-CoV-2 (MOI 1) during 48 h. **(A,B)** The levels of IFIT2 (an ISG), IFNL1 (a type III IFN), and CXCL10 (a pro-inflammatory cytokine) were evaluated by RT-qPCR, and mRNA levels were expressed as fold change (increases) in comparison to mock-treated cells, used as controls. Three different experiments were performed, with similar results. *p < 0.05 (using Student’s *t* test).

IAV and SARS-CoV-2 are unrelated viruses but in both cases viral replication is affected by IFN responses (Iwasaki and Pillai, 2014; Vanderheiden et al., 2020). Given that several ISGs, e.g., IFI6, IFI44 and IFI44L, negatively modulate IFN responses (DeDiego et al., 2019a,b; Villamayor et al., 2023), we postulated that IFI27 could play a role downregulating IFN-mediated host antiviral responses. To test this hypothesis, parental and IFI27 KO human A549 cells WT or expressing hACE2 were infected with IAV (MOI 1) or SARS-CoV-2 (MOI 1), respectively. Expression of IFN-induced protein with tetratricopeptide repeats (IFIT2, an ISG), IFNL1 (a type III IFN), and CXCL10 (a pro-inflammatory cytokine) were evaluated by RT-qPCR (Figure 4). After IAV and SARS-CoV-2 infection, expression of IFIT2, IFNL1, and CXCL10 was upregulated, as expected (Figures 4A,B). Notably, expression of these mRNAs was upregulated to a higher extent in IFI27 KO A549 and A549-hACE2 cells infected with IAV (Figure 4A) and SARS-CoV-2 (Figure 4B), respectively, compared to parental cells infected with the same viruses, suggesting that IFI27 expression negatively modulates IFN responses.

**Figure 5:**
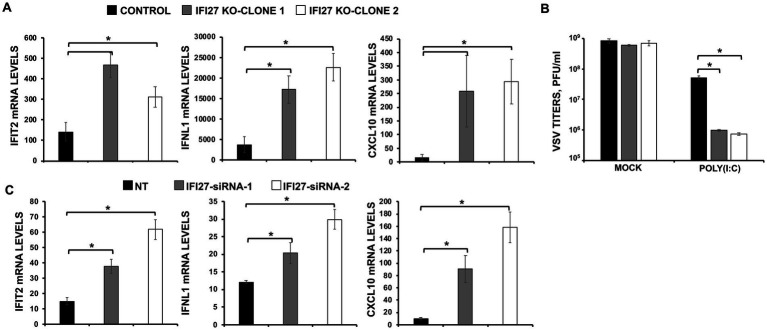
IFI27 impairs innate immune responses induced by poly(I:C). **(A,B)** Two clones of A549 cells specifically KO for the gene IFI27 were treated with poly(I:C). **(A)** The levels of IFIT2, IFNL1, and CXCL10 were evaluated by RT-qPCR at 24 hpt, and mRNA levels were expressed as fold change (increases) in comparison to mock-treated cells, used as controls. **(B)** Cells that had been subjected to mock treatment, or transfected with poly(I:C), were infected with rVSV-GFP (MOI of 0.1), and viral titers at 24 hpi were measured by a lysis plaque assay. Three different experiments were performed, with similar results. *p < 0.05 (using Student’s *t* test). **(C)** A549 cells were transfected with the NT siRNA or with two different siRNAs specific for IFI27. At 24 hpt, the cells were transfected with poly(I:C) during an additional 24 h. The levels of IFIT2, IFNL1, and CXCL10 were evaluated by RT-qPCR at 24 hpt, and mRNA levels were expressed as fold change (increases) in comparison to mock-treated cells, used as controls. Three different experiments were performed, with similar results. *p < 0.05 (using Student’s *t* test).

To confirm the role of IFI27 in negatively modulating IFN responses, parental and IFI27 KO A549 cells were transfected with poly(I:C). Expectedly (DeDiego et al., 2019a,b), transfection of parental cells with poly(I:C) induced the expression of IFIT2, IFNL1, and CXCL10 mRNA (Figure 5A) but the levels of IFIT2, IFNL1, and CXCL10 expression were upregulated to a higher extent in the two clones of IFI27 KO A549 cells encoding different deletions within the IFI27 open reading frame (ORF) (Figure 5A), further supporting the view that IFI27 negatively regulates induction of IFN responses.

To investigate whether IFI27’s ability to modulate IFN responses affects viral replication, parental human A549 cells or the two clones of IFI27 KO A549 cells were transfected with poly(I:C) to induce an antiviral state. Then, the cells were infected with a recombinant vesicular stomatitis virus expressing GFP, rVSV-GFP (MOI 0.1), as a indirect measure of the antiviral state induced by poly(I:C) transfection, given that VSV has been previously shown to be highly susceptible to the antiviral state induced by poly(I:C) and other IFN-inducible treatments (DeDiego et al., 2016; Nogales et al., 2017). At 24 hpi, rVSV-GFP grew with high titers (nearly 10^9^ pfu/ml) in parental and IFI27 KO A549 cells, which were mock-transfected with poly(I:C) (Figure 5B). However, virus titers decreased around 20-fold in poly(I:C)-transfected control A549 cells, negatively correlating with the induction of a host antiviral state in these cells (Figure 5B), as previously reported (DeDiego et al., 2019a,b). Notably, in IFI27 KO A549 cells transfected with poly(I:C), rVSV-GFP titers were around 60-fold lower than those in poly(I:C)-transfected control A549 cells (Figure 5B). These results further demonstrated that IFI27 expression negatively regulates the induction of host antiviral responses.

To further validate these results using another approach, expression levels of IFI27 in A549 cells were downregulated using the two different previously described siRNA treated with poly(I:C) and expression levels of IFIT2, IFNL1, and CXCL10 were determined by RT-qPCR. As shown with the IFI27 KO A549 cells, IFIT2, IFNL1 and CXCL10 were upregulated in siRNA IFI27-knocked-down A549 cells compared to NT-transfected control cells (Figure 5C), confirming our results with the KO A549 cells that IFI27 negatively modulates IFN responses.

**Figure 6:**
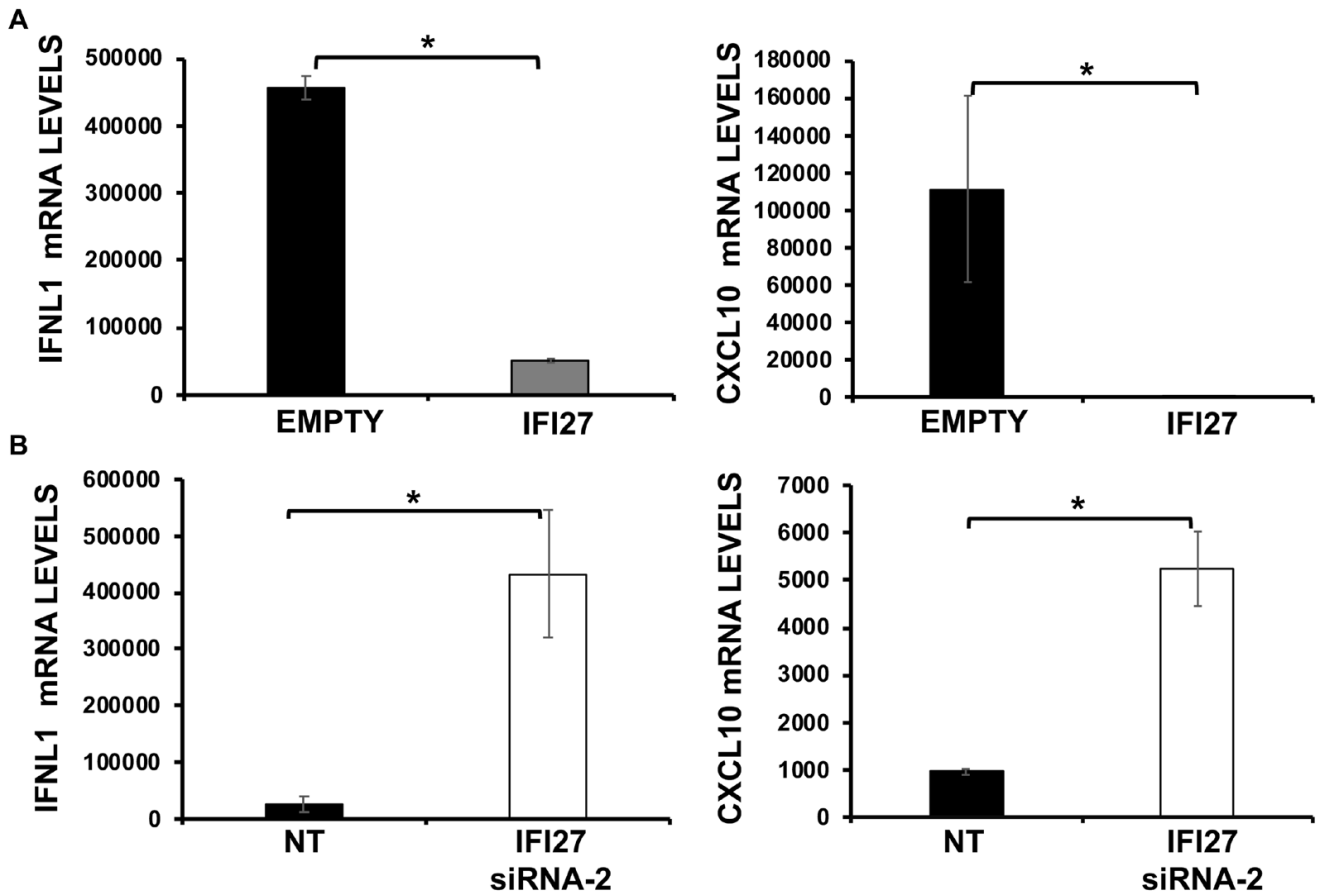
IFI27 impairs the induction of innate immune responses after SeV infection. **(A)** Human 293T cells were transfected with the plasmid pCAGGS-IFI27-HA or with the empty plasmid as control. **(B)** Alternatively, 293T cells were transfected with an IFI27 siRNA or with the NT siRNA, as control. **(A,B)** At 24 hpt, the cells were infected with SeV for an additional 24 h. The levels of IFNL1, and CXCL10 were measured by RT-qPCR and mRNA levels were expressed as fold change (increases) in comparison to mock-treated cells, used as controls. Three different experiments were performed, with similar results. *p < 0.05 (using Student’s *t* test).

We then investigated the effect of IFI27 in overexpression experiments. To that end, human 293T cells were transiently transfected with the pCAGGS plasmid expressing IFI27 fused to an HA tag, or with an empty pCAGGS plasmid, as control. Subsequently, cells were infected with SeV (MOI 3) and expression of IFNL1 and CXCL10 were determined by RT-PCR. Levels of IFNL1 and CXCL10 expression in control cells were increased (450,000 and 110,000-fold, respectively) whereas their expression was attenuated by 9 and 2,750-fold in cells transfected with the IFI27-encoding pCAGGS plasmid (Figure 6A). Conversely, and correlating with the overexpression data, induction of IFNL1 and CXCL10 in 293T cells infected with SeV was much higher (around 15 and 6-fold, respectively) in cells knocked-down for IFI27 expression than in the control cells transfected with the NT siRNA (Figure 6B). These results further confirmed that IFI27 negatively modulate IFN responses.

### Effect of IFI27 on viral replication and the induction of innate immune responses in mice

**Figure 7:**
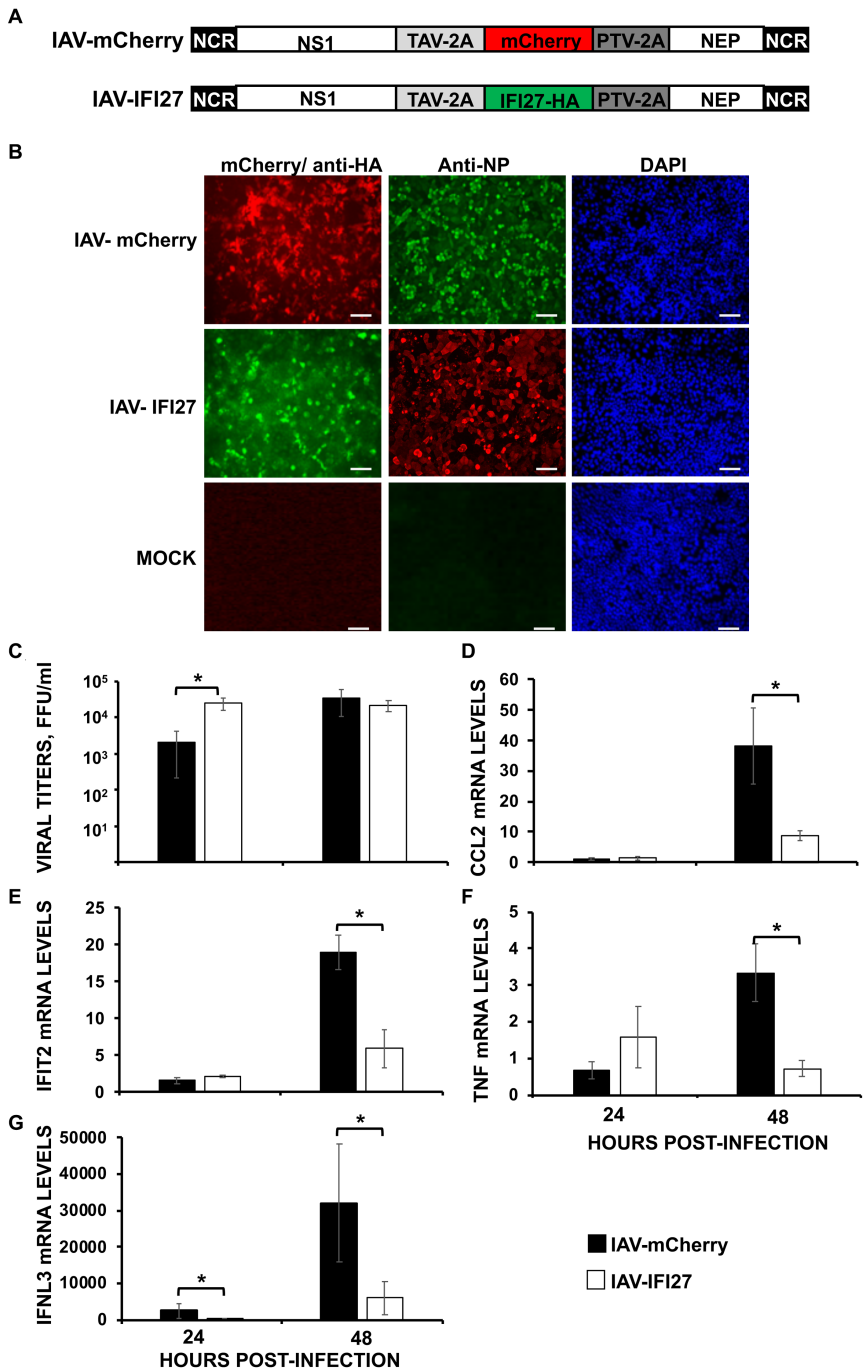
IFI27 expression negatively affects the induction of innate immune responses *in vivo*. **(A)** Schematic representation of PR8 viruses expressing mCherry or IFI27: A modified IAV-PR8 NS segment encoding NS1, mCherry (top) or IFI27-HA (bottom), and NEP are indicated. Black boxes at the beginning and end of each viral segment represent the viral 3′ and 5′ noncoding regions (NCR). White boxes indicate the viral NEP and NS1 proteins. The thosea asigna virus (TAV) 2A 2A autoproteolytic cleavage site used for the expression of NS1 and mCherry/IFI27 and the porcine teschovirus (PTV) 2A autoproteolytic cleavage site used for the expression of mCherry/IFI27 and NEP are indicated. **(B)** MDCK cells were non-infected (MOCK) or infected (MOI 0.1) with IAV-mCherry and IAV-IFI27. At 24 hpi, cells were fixed and permeabilized and visualized for mCherry expression. Then, the cells were stained with anti-HA and anti-NP Abs. DAPI was used for nuclear staining. Representative images (20× magnification) are included. Scale bar, 50 μm. **(C–G)** Mice (*n* = 4/group) were infected with IAV-mCherry and IAV-IFI27 viruses (2,000 FFU/mice). **(C)** At 24 and 48 h post-infection, viral titers in infected lungs were determined. **(D–G)** At 24 and 48 h post-infection, CCL2 **(D)**, IFIT2 **(E)**, TNF **(F)**, and IFNL3 **(G)** expression was evaluated in mice lungs by RT-qPCR. mRNA levels were expressed as fold change (increases) in comparison to mock-infected mice, used as controls. *p < 0.05 (using Student’s *t* test).

To further analyze whether IFI27 confers a significant role on affecting viral replication and on counteracting IFN responses *in vivo*, recombinant IAVs expressing IFI27 and mCherry (as control) in their genomes were generated (IAV-IFI27 and IAV-mCherry, respectively). To this end, IFI27 and mCherry ORFs were cloned in plasmids encoding an IAV NS split segment, so that the NS1, NEP and IFI27/mCherry proteins were autoproteolytically processed as they were flanked by thosea asigna virus (TAV) 2A autoproteolytic site, between NS1 and IFI27 or between NS1 and mCherry, and flanked by the porcine teschovirus (PTV) 2A autoproteolytic site, between IFI27 and NEP or mCherry and NEP, respectively (Figure 7A). To demonstrate that viruses expressed the foreign IFI27 and mCherry proteins, MDCK cells were mock-infected or infected (MOI 0.1) with IAV-mCherry and IAV-IFI27, and expression of mCherry and IFI27 were determined by fluorescent and immunofluorescence, respectively. In addition, we performed immunofluorescence of infected cells using an antibody specific for the viral NP to demonstrate similar levels of viral infection. The results showed that most infected cells (NP antibody) also expressed mCherry and IFI27-HA (Figure 7B).

Next, groups of C57BL/6 mice were infected intranasally with the recombinant viruses (2,000 FFU/mice) and viral titers were analyzed in mouse lungs at 24 and 48 hpi. Correlating with data shown in Figure 2C, significant differences in viral titers were observed at 24 hpi, being titers higher in mice infected with the IFI27-expressing virus as compared to IAV-mCherry-infected mice (Figure 7C). However, no differences were observed at 48hpi suggesting a role of IFI27 in viral replication early during infection. In addition, we assessed levels of IFIT2, IFNL3 and typical pro-inflammatory cytokines (TNF) and chemokines (CCL2) at 24 and 48 hpi (Figures 7D–G). At 24 hpi, the expression of TNF, CCL2, and IFIT2 was not significantly increased in infected mice as compared to mock-infected mice (Figures 7D–F), and expression of IFNL3 was upregulated to a limited degree (Figure 7G). Contrary, we observed a clear upregulation of TNF, CCL2, IFIT2, and IFNL3 in the infected mouse lungs at 48 hpi. Remarkably, at 48 hpi, expression of TNF, CCL2, IFIT2, and IFNL3 was significantly upregulated in the lungs of mice infected with IAV-mCherry compared to the lungs of IAV-IFI27-infected mice (Figures 7D–G). In addition, expression of IFNL3 was significantly higher in the lungs of IAV-mCherry-infected mice compared to IAV-IFI27-infected mice at 24 hpi (Figure 7G). These results in mice are in agreement with the *in vitro* data shown in Figures 2–5, and further demonstrate that IFI27 counteracts innate immune responses not only *in vitro* but also *in vivo*. In addition, our results suggest that recombinant IAV expressing human genes represent a valid and novel strategy to study the role of host cellular factors in viral infection and in ISG responses *in vivo*.

### Molecular mechanism of action of IFI27

**Figure 8:**
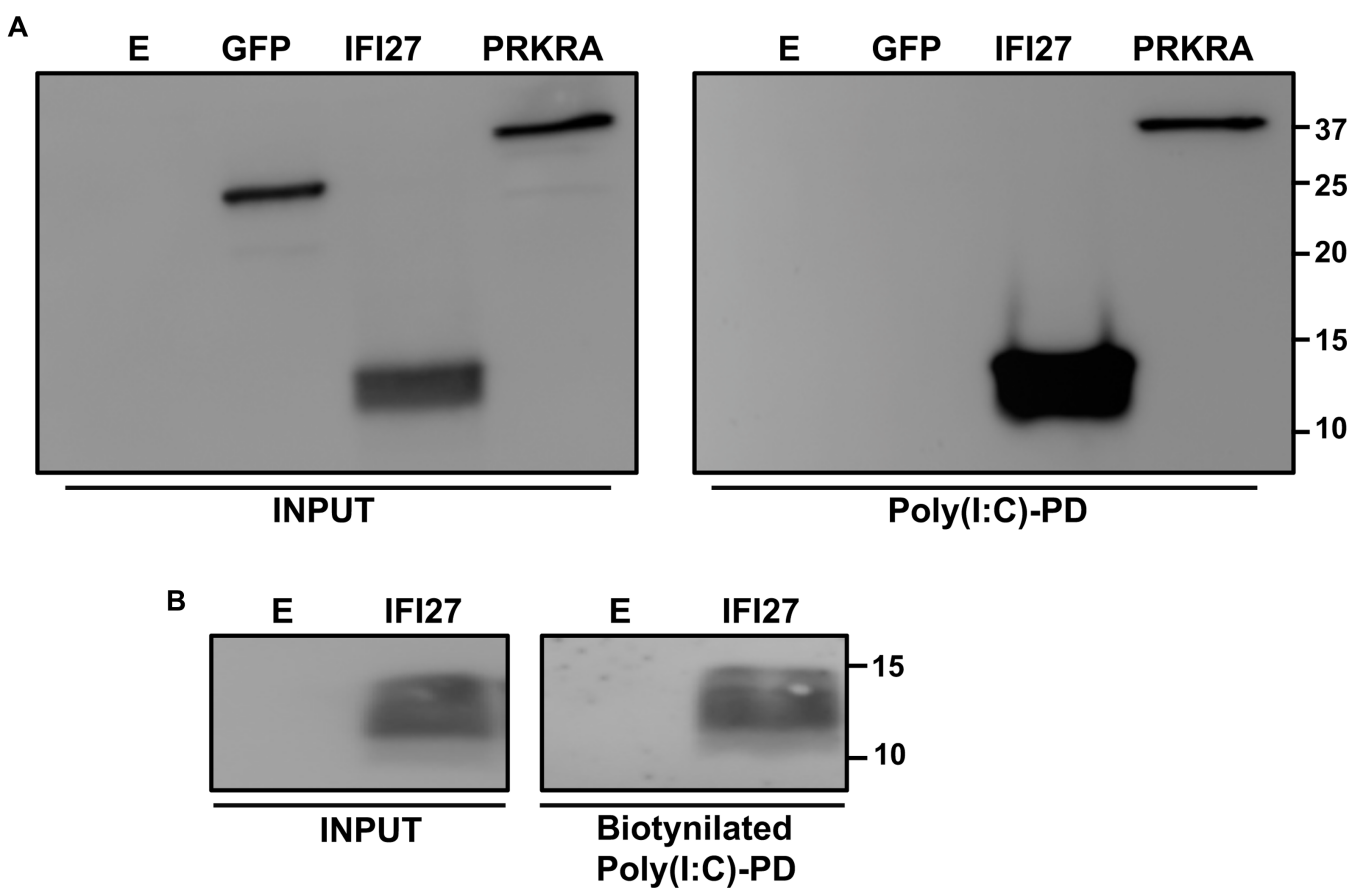
Binding of IFI27 to poly(I:C). **(A)** Human 293T cells were transiently transfected with the pCAGGS plasmids encoding GFP, IFI27-HA and PRKRA-FLAG, or with an empty plasmid. Pull-down (PD) experiments using poly(C) (data not shown) and poly(I:C)-conjugated agarose beads were performed using cellular extracts. Western blotting using antibodies specific for GFP, the HA tag (to detect IFI27) or the FLAG tag (to detect PRKRA) was performed to detect protein in the cellular lysates (Input) and after the pull-down (poly(I:C)-PD). Molecular weight markers are indicated (in kilodaltons) on the right. **(B)** Human 293T cells were transiently transfected with the pCAGGS-IFI27-HA or with an empty plasmid. Then, the cells were transfected with poly(I:C) conjugated to biotin or with unconjugated poly(I:C), as control (data not shown). The cellular extracts were incubated with agarose beads bound to streptavidin. Western blotting using antibodies specific for the HA tag (to detect IFI27) was performed to detect protein in the cellular lysates (Input) and after the pull-down (biotinylated poly(I:C)-PD). Molecular weight markers are indicated (in kilodaltons) on the right.

According to bioinformatic predictions using RNABindRplus, a method that combines machine learning and sequence homology-based approaches to improve the reliability of predicted RNA-binding residues in proteins (Walia et al., 2014, 2017), IFI27 contains 13 amino acids which are predicted to bind RNA, including, specifically, the amino acids 60–65, 68, 69, and 82–86. Therefore, IFI27 could negatively modulate innate immune responses through binding to viral RNAs and poly(I:C). To test this hypothesis, cells were transfected with the pCAGGS plasmid expressing IFI27 fused to an HA tag, or with pCAGGS plasmids expressing GFP or PRKRA, a dsRNA-binding protein that was used as a positive control (Patel and Sen, 1998). Then, cellular lysates were exposed to agarose beads conjugated to either poly(I:C), an analog of dsRNA (Figure 8A), or poly(C) as control (data not shown). Remarkably, IFI27 bound the poly(I:C)-conjugated agarose beads, but not the control poly(C)-conjugated agarose beads. As expected, GFP, did not bind poly(I:C) or poly(C)-agarose beads, whereas PRKRA bound to agarose beads conjugated with poly(I:C) but not poly(C) (Figure 8A and data not shown) (Patel and Sen, 1998). To confirm the binding of IFI27 to poly(I:C) using a different approach, cells were transfected with the pCAGGS plasmid expressing IFI27, and then, cells were transfected with poly(I:C) conjugated or not conjugated (as control) to biotin. The cellular extracts were then bound to agarose beads conjugated to streptavidin, and the presence of IFI27 was analyzed by Western blot. IFI27 bound to streptavidin-conjugated agarose beads, when the cells were transfected with biotinylated poly(I:C) (Figure 8B). As control, IFI27 did not bind to streptavidin-conjugated agarose beads when the cells were transfected with non-biotinylated poly(I:C) (data not shown), further showing that IFI27 binds poly(I:C), and likely RNAs.

**Figure 9:**
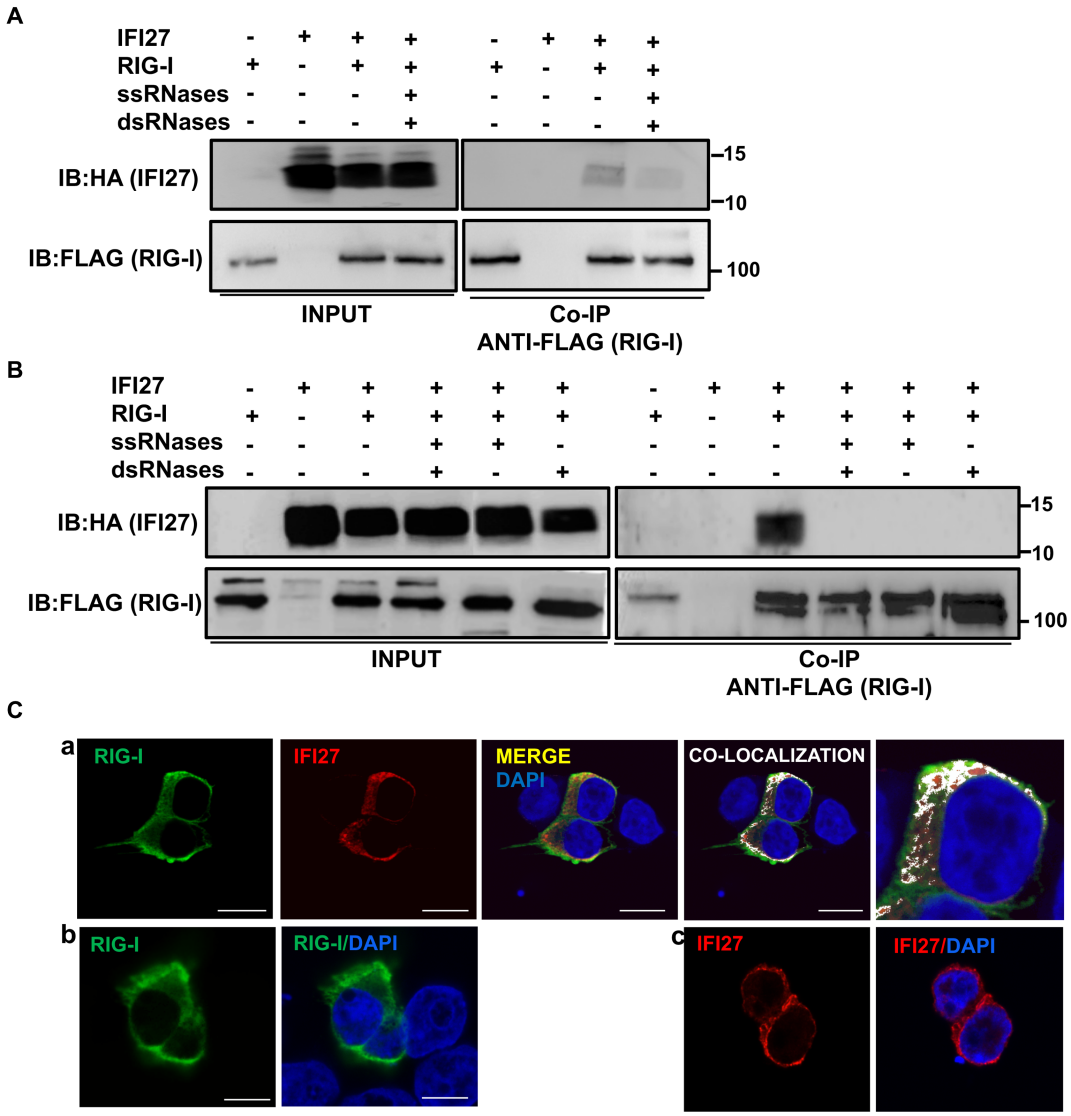
IFI27 binds RIG-I. **(A–C)** Human 293T cells were transiently co-transfected with the pCAGGS plasmids encoding IFI6-HA and RIG-I-FLAG, or with empty plasmids. At 24 hpt, the cells were transfected with poly(I:C) during 24 h **(A,C)** or infected with SeV for 24 h **(B)**. **(A,B)** Cellular extracts were either treated with RNases digesting ssRNAs (ssRNases), RNases digesting dsRNAs (dsRNases), or non-treated with RNases and co-immunoprecipitation (Co-IP) experiments using an anti-FLAG antibody, to pull down RIG-I were performed. IFI27 and RIG-I were detected by Western blotting using antibodies specific for the HA tag (to detect IFI27, top panels) or the FLAG tag (to detect RIG-I protein, bottom panels) in the cellular lysates (Input) and after the Co-IP. IB, immunoblot. Molecular weight markers (in kilodaltons) are indicated on the right. (C) At 24 hpt, cells were fixed with paraformaldehyde, and RIG-I-FLAG and IFI27-HA were labeled with antibodies specific for the tags (in green and red, respectively), and nuclei were stained with DAPI (in blue). Pictures show cells co-transfected with plasmids expressing RIG-I and IFI27 together (a), and cells transfected with the plasmids expressing RIG-I (b) and IFI27 (c) separately. (a) Areas of co-localization of both proteins appear in yellow in the third picture and in white in the fourth picture. A zoom of the colocalization image (from fourth picture) is depicted in the fifth picture. Scale bar, 10 μm.

Based on these results, we postulated that IFI27 could bind RIG-I, through binding to RNA, given that RIG-I has been shown to bind ssRNAs and dsRNAs (Pichlmair et al., 2006; Thoresen et al., 2021) and that SARS-CoV-2, IAV and SeV infection get recognized by RIG-I in infected cells (Kato et al., 2006; Rehwinkel et al., 2010; Kouwaki et al., 2021; Wu et al., 2021; Marx et al., 2022). To test this hypothesis, we transfected 293T cells with pCAGGS plasmids expressing IFI27 fused to the HA tag and RIG-I fused to a FLAG tag. Then, we transfected cells with poly(I:C) and cell extracts were collected and immunoprecipitated using agarose beads conjugated to a FLAG antibody. Interestingly, RIG-I and IFI27 co-immunoprecipitated together (Figure 9A). Moreover, IFI27 was not detected when RIG-I was not overexpressed, suggesting a direct or indirect interaction of IFI27 and RIG-I. As RIG-I (Rehwinkel and Gack, 2020) and IFI27 (Figure 8) both bind poly(I:C), we next analyzed whether the interaction of IFI27 and RIG-I was mediated by RNA by performing immunoprecipitation under the same experimental conditions using cell extracts treated with RNAseT1 and RNaseA (to digest ssRNAs), and with RNAseIII (to digest dsRNAs) (Figure 9A). While the amount of IFI27 protein in the cellular extracts before the immunoprecipitation was similar irrespective of the RNase treatment (Figure 9A), the amount of IFI27 protein co-immunoprecipitated with RIG-I was clearly decreased, almost to undetectable levels, in the cell extracts previously treated with both RNAses. These results strongly suggest that IFI27 with RIG-I is RNA-mediated. To further confirm this RNA-mediated interaction of IFI27 with RIG-I, similar experiments were performed using cell extracts from SeV-infected cells (Figure 9B). Again, IFI27 interacted with RIG-I, and this interaction was significantly affected when the cell extracts were treated with RNAseA and RNaseT1 to digest single-stranded (ss)RNAs, with RNAseIII to digest dsRNAs, or with the three RNAses together (Figure 9B), confirming that the interaction of IFI27 with RIG-I is mediated by RNAs, suggesting the data that the interaction could be mediated by ssRNA and/or dsRNA.

To study whether IFI27 and RIG-I colocalize intracellularly, 293T cells were transiently transfected with the pCAGGS plasmids expressing the tagged versions of RIG-I and IFI27, and then transfected with poly(I:C). We observed a partial co-localization of IFI27 and RIG-I in distal regions of the cytoplasm (Figure 9C), as analyzed by immunofluorescence and confocal microscopy, reinforcing the interaction of IFI27 and RIG-I from the co-IP experiments.

**Figure 10:**
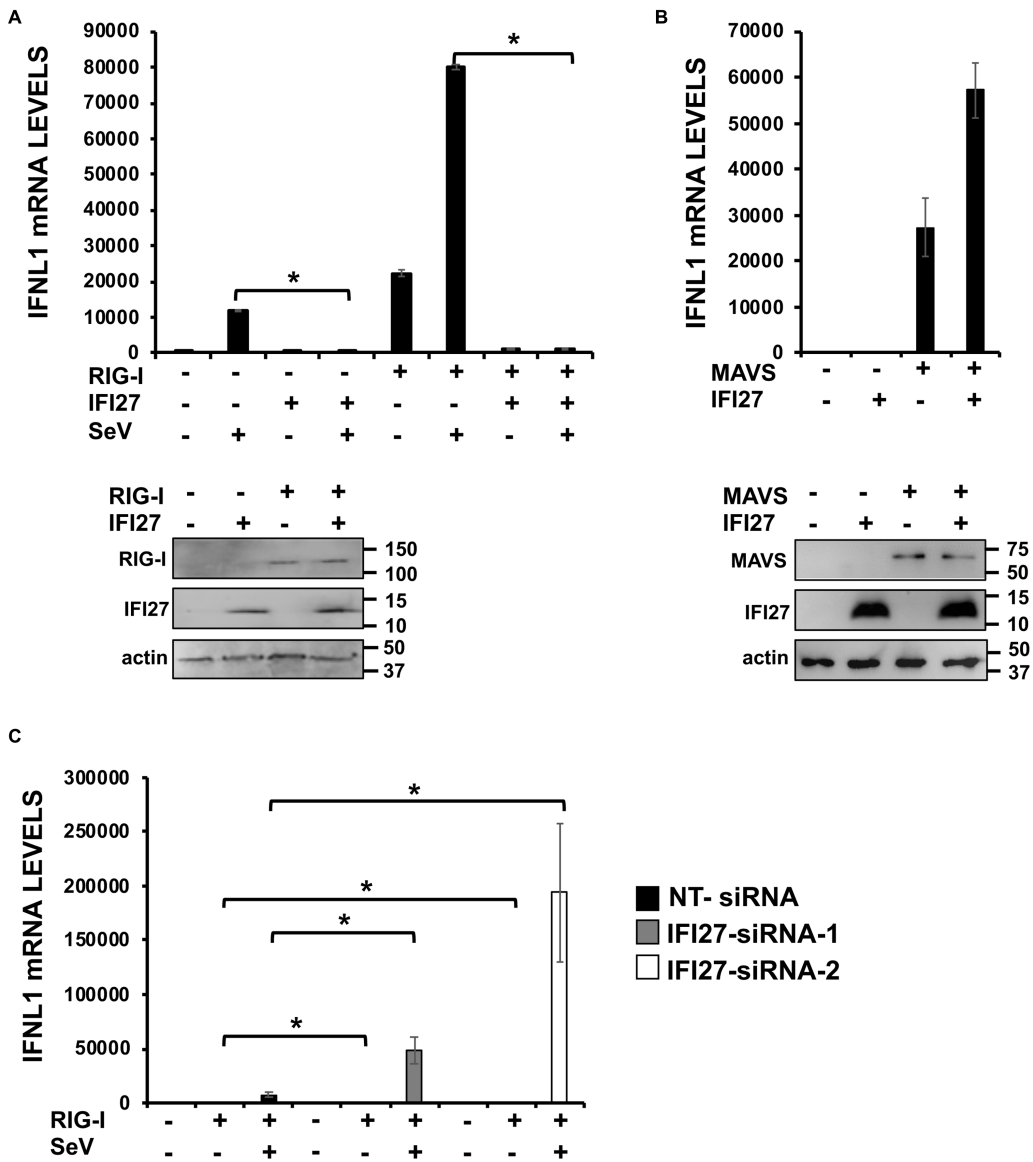
IFI27 impairs RIG-I activation. **(A)** Human 293T cells were transiently co-transfected with the pCAGGS plasmids encoding IFI27-HA and RIG-I-FLAG, or with empty plasmids. The cells were infected with SeV (MOI 3) during 24 h. **(B)** Human 293T cells were transiently co-transfected with the pCAGGS plasmids encoding IFI27-HA and MAVS-FLAG, or with empty plasmids. **(A,B)** Proteins in the cellular extracts from non-infected cells were subjected to Western blotting using antibodies specific for FLAG tag (to detect RIG-I protein in **A** or MAVS in **B**), the HA tag (to detect IFI27 in **A,B**) or actin (in **A,B**), as loading control. **(C)** Human 293T cells were transfected with two siRNAs specific for IFI27 or with the NT siRNA and 24 h after siRNA-transfections, the cells were transfected with the pCAGGS plasmid encoding RIG-I-FLAG, or with the empty plasmid, as control. Then, the cells were infected with SeV (MOI 3) during 24 h. **(B,C)** Total RNAs were extracted and the levels of IFNL1 were measured by RT-qPCR and mRNA levels were expressed as fold change (increases) in comparison to control cells, used as controls. Three different experiments were performed, with similar results. *p < 0.05 (using Student’s *t* test).

To confirm that the interaction of IFI27 with RIG-I results in IFI27 negatively modulating the induction of innate immune responses mediated by RIG-I, 293T cells were transiently co-transfected with the pCAGGS plasmid expressing RIG-I-FLAG and IFI27-HA. The expression of both proteins was confirmed by Western blot using anti-HA (to detect IFI27) and anti-FLAG (to detect RIG-I) specific antibodies (Figure 10A). Then, the cells were either infected with SeV or left uninfected, as control (Figure 10A). According to previous results (Osterlund et al., 2007), overexpression of RIG-I induced the expression of IFNL1 (~ 20,000-fold, Figure 10B) in the absence of any viral infection. Interestingly, expression of IFNL1 was further induced in cells overexpressing RIG-I after SeV infection (~ 80,000-fold, Figure 10B). Importantly, overexpression of IFI27 led to decreased IFNL1 induction in RIG-I overexpressing mock-infected or SeV-infected cells (Figure 10A). Notably, when the levels of IFNL1 were induced by overexpression of mitochondrial antiviral signaling protein (MAVS), an scaffold adaptor involved in RIG-I activation (Belgnaoui et al., 2011), IFI27 overexpression had minimal or no effect on the levels of IFNL1 (Figure 10B). Expression levels of MAVS and IFI27 were confirmed by Western blot using antibodies specific for FLAG (MAVS) and HA (IFI27) (Figure 10B). These data suggest that the effect of IFI27 in negatively modulating RIG-I activation is specific, as it does not affect MAVS activation.

To further corroborate these results, 293T cells were silenced for IFI27 using the two previously described siRNAs. Then, cells were transiently transfected with the pCAGGS plasmid expressing RIG-I-FLAG, and cells were either mock-infected or infected with SeV (Figure 10C). Expression of IFNL1 mRNA was higher in knocked-down IFI27 cells compared to NT siRNA transfected cells overexpressing RIG-I, irrespective of whether the cells were mock-infected or infected with SeV (Figure 10C). These results strongly suggested that IFI27 modulates activation of RIG-I.

## Discussion

This work provides evidence for a novel function of IFI27, an ISG, in impairing innate immune responses mediated by different and unrelated viruses, including IAV, SARS-CoV-2, and SeV, as well as in cells transfected with poly(I:C). In addition, we show that blocking IFI27 expression negatively correlates with viral titers, an effect most likely due to IFI27’s ability to counteract innate immune responses. Moreover, we show that the underlying mechanism involves the ability of IFI27 to bind dsRNA and RIG-I, being the interaction of IFI27 and RIG-I mediated by binding of IFI27 to RNA. In addition, we provide evidence showing that interaction of IFI27 with RIG-I affects RIG-I activation. Although innate immune responses are beneficial to combat viral infections, exacerbated inflammatory responses after viral infections are detrimental to the host (Komuro et al., 2008; Richards and Macdonald, 2011), so, in this sense, IFI27 could prevent exacerbated innate immune responses to viral infections.

Expression of IFI27 is induced after infection with different viruses. For example, high levels of IFI27 expression have been detected in blood samples of infants hospitalised with RSV (Fjaerli et al., 2006), influenza virus-infected patients (Tang et al., 2017; Ravi et al., 2022), and SARS-CoV-2 positive patients (Gupta et al., 2021; Huang et al., 2021). In addition, high levels of IFI27 expression were associated with enhanced severity of RSV infection (Gao et al., 2021). Accordingly, we show that in cell culture, IFI27 is induced after IAV and SARS-CoV-2 infections (Figures 1B,C).

Decreasing IFI27 expression negatively affects viral production (Figures 2C,D, 3C), and IFI27 counteracts host antiviral responses not only *in vitro* (Figures 4–6) but also *in vivo* (Figures 7D–G). Furthermore, we observe decreased VSV titers in poly(I:C)-transfected cells knocked-out for IFI27 compared to parental poly(I:C)-transfected control cells (Figure 5B). The effect of IFI27 facilitating viral replication is likely mediated by negative modulation of innate immune responses as suggested by experiments performed taking advantage of the fact that the response to VSV infection is affected by the previous antiviral states induced in the cells (DeDiego et al., 2016; Nogales et al., 2017). Similarly, we previously showed that IFI6, IFI44 and IFI44L counteract innate immune responses and positively affect IAV and coronavirus replication (DeDiego et al., 2019a,b; Villamayor et al., 2023), and silencing other ISGs such as IFI35 and ISG56/IFIT1 proteins, which negatively modulate IFN responses, decreases VSV replication (Li et al., 2009; Das et al., 2014). Many ISGs induced after viral infections display antiviral activities, however, other ISGs counteract innate immune responses, supporting virus replication, and providing negative feedback mechanisms, since exacerbated immune responses are deleterious to the host (Komuro et al., 2008; Richards and Macdonald, 2011).

Notably, we have shown that by overexpressing IFI27 in the IAV genome, the cellular functions as well as the effect of IFI27 on viral replication can be studied *in vivo*. These results suggest the feasibility of using this novel expression strategy to study the function(s) of other host factors, including their function and contribution to viral replication *in vitro*, without the need to genetically manipulate the experimental animals.

We describe a novel interaction of IFI27 with poly(I:C), an analog of dsRNA (Figure 8). Moreover, we show that IFI27 binds RIG-I, using both poly(I:C)-transfected cells and SeV-infected cells (Figures 9A,B), and that IFI27 and RIG-I partially colocalize intracellularly (Figure 9C). In addition, we show that the interaction of IFI27 with RIG-I is most likely mediated by RNAs (Figures 9A,B, respectively). After ssRNA and dsRNA recognition, RIG-I interacts with MAVS, also known as cardif, IPS-1 and VISA, favoring the activation of the transcription factors IRF-3, IRF-7 and NF-κB (Meylan et al., 2005; Seth et al., 2005), which lead to the expression of multiple proinflammatory cytokines, IFNs and ISGs (Meylan et al., 2005; Seth et al., 2005). Therefore, as IFI27 is a protein localized to the mitochondria (Cheriyath et al., 2011; Jin et al., 2018), such as RIG-I and MAVS, these data favor an interaction of IFI27 with RIG-I (Figures 9A,B).

RIG-I recognizes viral RNAs generated during infection with different viruses, including SARS-CoV-2 (Kouwaki et al., 2021; Mao et al., 2022; Marx et al., 2022), IAV (Kato et al., 2006; Rehwinkel et al., 2010), and SeV [35], as well as transfected poly(I:C) (Dauletbaev et al., 2015), being its activation tightly regulated (Brisse and Ly, 2019). In fact, the therapeutic treatment with RIG-I agonists prevented infection with SARS-CoV-2 in an IFN-dependent manner (Mao et al., 2022) and the prophylactic and therapeutic treatment with another RIG-I agonist improved survival after SARS-CoV-2 infection by preventing viral replication and inflammation (Marx et al., 2022). RIG-I is expressed endogenously in the cytoplasm of the cells in an inactivated conformation. However, upon recognition of dsRNA, RIG-I is activated. In this work, we show that IFI27 binds poly(I:C) (Figure 8), suggesting that this interaction may impair the binding of RIG-I to poly(I:C) that this interaction impairs the binding of RIG-I to poly(I:C), therefore preventing its activation (Figure 10A). In contrast, other host proteins, as the helicases DDX6, DHX15, DHX29, and DDX60 have been identified as RIG-I co-factors that interact with RIG-I and with viral RNAs and dsRNAs to increase RIG-I activity (Miyashita et al., 2011; Sugimoto et al., 2014; Núñez et al., 2018; Pattabhi et al., 2019; Hage et al., 2022). However, the interaction of DHX29 and DDX60 with RIG-I seems independent of RNAs (Miyashita et al., 2011; Sugimoto et al., 2014), as oppose to the interaction of IFI27 with RIG-I. Similarly, IFI16 also interacts with RIG-I, increasing RIG-I transcription and activation after IAV infections (Jiang et al., 2021). Many of these proteins regulating RIG-I activation, also affect viral replication, i.e., DDX60, DHX15, DHX16 and DDX6, which increase RIG-I activation, negatively affect VSV and poliovirus (Miyashita et al., 2011), encephalomyocarditis virus (EMCV) (Pattabhi et al., 2019), and IAV, Zika virus, SARS-CoV-2 (Hage et al., 2022), and enterovirus (Zhang et al., 2021) replication, respectively.

In addition to RIG-I, other cellular proteins, such as MDA-5 and TLR-3, detect viral RNAs, initiating IFN downstream responses (Gürtler and Bowie, 2013; Brisse and Ly, 2019). Whether or not IFI27 affects the activation of these other host factors, and thus, induction of innate immune responses mediated by these other PRRs remains uncovered and guarantee further investigation.

## Data availability statement

The original contributions presented in the study are included in the article/supplementary material, further inquiries can be directed to the corresponding author.

## Ethics statement

Procedures involving animals were approved by the CSIC Ethics Committee for animal experimentation and by the Division of Animal Protection of the Regional Government of Madrid in compliance with National and European Union legislation (PROEX89.5/20).

## Author contributions

LV, DL-G, and VR: investigation, methodology, validation, formal analysis, data curation, and writing – review and editing. LM-S: conceptualization, resources, and writing – review and editing. AN: conceptualization, investigation, methodology, validation, formal analysis, data curation, and writing – review and editing. MD: conceptualization, resources, investigation, methodology, validation, formal analysis, data curation, writing – original draft, writing – review and editing, visualization, supervision, and funding acquisition. All authors contributed to the article and approved the submitted version.

## Funding

This work was supported by MCIN/AEI/10.13039/501100011033/FEDER, UE (PID-2021-123810OB-I00) and AEI/10.13039/501100011033 (RTI-2018-094213-A-I00) to MD, the “Atracción de Talento Investigador” programme (2017-T1/BMD-5155) funded by the “Comunidad de Madrid” to MD, the European Commission – NextGenerationEU (Regulation EU 2020/2094), through Spanish National Research Council (CSIC)’s Global Health Platform (PTI Salud Global, CSIC-COV19-012/012202020E086) to MD, and a “Ramon y Cajal” Incorporation grant (RYC-2017) from the Spanish Ministry of Science, Innovation and Universities to AN. The project that gave rise to these results received the support of a fellowship from “la Caixa” Foundation (ID 100010434). The fellowship code is LCF/BQ/DR22/11950020 (to DLG)”

## Conflict of interest

The authors declare that the research was conducted in the absence of any commercial or financial relationships that could be construed as a potential conflict of interest.

## Publisher’s note

All claims expressed in this article are solely those of the authors and do not necessarily represent those of their affiliated organizations, or those of the publisher, the editors and the reviewers. Any product that may be evaluated in this article, or claim that may be made by its manufacturer, is not guaranteed or endorsed by the publisher.

## Footnotes

^1^https://www.atum.bio/eCommerce/cas9/input?multipleContacts=false and http://crispor.tefor.net
